# Relationship between diabetes health literacy, distress, burnout, social support, complications, self-care behaviors, and quality of life among patients with type 2 diabetes: a path analysis study

**DOI:** 10.1186/s13098-024-01391-z

**Published:** 2024-07-05

**Authors:** Alireza Jafari, Fatemehzahra Naddafi^, Mahdi Gholian‑Aval, Hadi Tehrani

**Affiliations:** 1 Department of Health Education and Health Promotion, School of Health, Social Development and Health Promotion Research Center, Gonabad University of Medical Sciences, Gonabad, Iran; 2 Student Research Committee, Gonabad University of Medical Sciences, Gonabad, Iran; 3 Social Determinants of Health Research Center, Mashhad University of Medical Sciences, Mashhad, Iran; 4 Department of Health Education and Health Promotion, School of Health, Mashhad University of Medical Sciences, Mashhad, Iran

**Keywords:** Diabetes, Burnout, Distress, Quality of life, Self-care, Social support, Health literacy

## Abstract

**Introduction:**

Improving the quality of life (QOL) is the most important goal of early diagnosis and treatment in patients with type 2 diabetes (T2D). Numerous studies have indicated the positive effects of health literacy, social support and self-care behaviors and the negative effects of diabetes distress and burnout on the QOL of patients with T2D. Understanding these factors is crucial for people with diabetes. However, no study has investigated the simultaneous effects of these variables on QOL. In this study, our goals were to find out how these variables are related to each other, in addition, which variables play the role of mediating variables, and finally, what is the cumulative effect of these variables in predicting the QOL of patients with T2D. So, this study aimed to examine the relationship between diabetes health literacy (DHL), distress, burnout, social support, complications of diabetes, self-care behaviors, and QOL among patients with T2D by application Path analysis method.

**Methods:**

In this study 929 participants were entered to study by cluster sampling method and finally, data were analyzed among 820 participants. Data were gathered by self-report and with seven tools of Demographic section, DHL Scale, Diabetes distress scale, Diabetes Burnout scale, Diabetes Self-Management Questionnaire (DSMQ), Perceived social support, Diabetes Quality of Life (DQOL) Questionnaire. The software’s of SPSS version 24 and AMOS version 24 were used for analysis.

**Results:**

The variables of DHL, social support, diabetes distress, and complications of diabetes predicted 38% variance in diabetes burnout (R^2^ = 0.38). Greatest impact on diabetes burnout was related to diabetes distress (estimate total effect = 0.539). The variables of DHL, social support, diabetes distress, complications of diabetes, and diabetes burnout predicted 24% variance in self- care behaviors (R^2^ = 0.24). Greatest impact on self- care behaviors was related to DHL (estimate total effect = 0.354). The variables of DHL, social support, diabetes distress, diabetes burnout, complications of diabetes, and self- care behaviors predicted 49% variance in DQOL (R^2^ = 0.49). Greatest impact on DQOL was related to variables of diabetes distress (estimate total effect = -0.613), DHL (estimate total effect = 0.225), diabetes burnout (estimate total effect = -0.202), complications of diabetes (estimate total effect = − 0.173), social support (estimate total effect = 0.149), and self -care (estimate total effect = 0.149), respectively.

**Conclusion:**

To improve QOL in patients with T2D, health care providers must develop interventions that increase DHL of diabetic. Because DHL can decrease distress and burnout, enhance self -care skills, create supportive networks, and ultimately improve QOL in patients with type 2 diabetes.

## Introduction

Type 2 diabetes (T2D) is the global challenge of the 21st century and the most common metabolic disease [1, 2]. This type of diabetes, which is associated with insufficient accountability to insulin, is now known as modern pandemic [3–5]. According to the International Diabetes Federation, 537 million (one out of every ten) of adults in 2021 had diabetes and is expected to increase to 643 and 783 million in 2030 and 2045, respectively [6]. The prevalence of diabetes in Iran was also reported in 2021 in people over 18 years old, which was 45.5% increase compared to 2016 [7] and by 2030, about 9.2 million Iranians are expected to have diabetes [8].

The main purpose in early diagnosis, treatment and care interventions is to maintain and improve the quality of life (QOL) [9, 10]. QOL is referred to as one’s understanding of one’s physical, social, and mental state, one’s sense of self, and overall satisfaction with life [11, 12]. In fact, diabetes leads to decrease QOL in various aspects of physical (by increasing cardiovascular disease, stroke, neuropathy, etc.), psychological (with increased emotional distress, depression) and social (such as lifestyle changes, loss Occupation, increases costs) [13–16]. As a result, people with diabetes have lower QOL than healthy people [11, 17]. The systematic review and meta -analysis study found that Iranian diabetic also have lower QOL than the normal population [18]. The QOL of people with diabetes is influenced by a complex and multifaceted interaction of various factors [13, 19, 20]. Understanding and evaluating these factors can be helpful in improving health and enhancing QOL of those with diabetes [15, 21]. In recent studies, variables such as diabetes health literacy (DHL), social support, diabetes distress, diabetes burnout, complication, and self -care have been identified as factors affecting QOL [22–27].

DHL refers to the ability and skill of people with diabetes in search, understanding, analysis of diabetes information to manage and treat their disease [28, 29]. Based on the results of studies, a significant relationship was found between DHL and QOL of people [22, 30]. A study in Iran also found that DHL had more effect on improving QOL of patients [13]. Perceived social support is one of the effective factors in QOL and refers to the instrumental support, informational support, and emotional support provided by family, friends and other people to help diabetic [31–33]. The findings of the study showed a significant relationship between social support and QOL among T2D [23]. Social support can also promote DHL in patients [29, 34].

Diabetes distress is a negative emotional reaction in which people with diabetes experience emotions such as fear, despair, sin, stress, worry or denial, and this is due to the burden of permanent life with diabetes and self -management behaviors [35, 36]. The results of cross -sectional study in Iran showed that 47% of T2D suffered from diabetes distress [37]. The negative relationship between diabetes distress and QOL has been shown in numerous studies. In fact, diabetes distress is associated with a decrease in QOL [24, 38, 39]. Also, in another study among patients with diabetes distress, the negative relationship between distress, health literacy (HL) and social support was identified [40]. If diabetes distress is ignored and not treated, it can lead to diabetes burnout [41, 42]. Diabetes burnout refers to the feeling of severe physical, mental, and emotional fatigue caused by diabetes [41, 42]. Diabetes burnout is actually a combination of behaviors and feelings, including detachment, exhaustion, and feeling powerless [43, 44]. Diabetes burnout is a serious obstacle in glycemic control and treatment adherence, which can cause neglect and even incompatibility with self-care behaviors and increase diabetes complications [42, 43]. In a study in patients with T2D, it was found that among 36% of them, diabetes burnout was an obstacle to medication adherence [45]. Diabetes burnout is another effective factor in reducing QOL. There is also a link between diabetes burnout and QOL among people with diabetes [25] but, as far as we know, this relationship has rarely been carefully investigated, especially in patients with T2D.

Finally, self -care refers to the ability of communities, families and individuals to maintain and promote health, prevent disease and manage disability and disease with or without health care [46]. Higher self -care behaviors can enhance QOL of those with T2D [26]. In a cross -sectional study in Iran, self -care behaviors had significant relationship with QOL of patients with T2D [47]. On the other hand, lower QOL will also can lead decrease the self -care behaviors [48]. A study in patients with diabetes also found that there was a strong relationship between DHL and self -care behaviors [49]. Another study found that there is a negative relationship between diabetes distress and self -care behaviors [50]. Social support as an essential element also plays an important role in facilitating and enhancing self -care behaviors [51, 52], while diabetes burnout can neglect self -care behaviors [41]. Based on the search in the literature review, a study that simultaneously examined these variables and their relationship with self-care behaviors and QOL of people with diabetes was not observed.

The first hypothesis of this study was that DHL, diabetes distress, complications of diabetes, and social support had direct and indirect effect on diabetes burnout among patients with T2D. The second hypothesis of this study was that DHL, diabetes distress, complications of diabetes, diabetes burnout, and social support had direct and indirect effect on self -care behaviors among patients with T2D. The third hypothesis of this study was that DHL, diabetes distress, diabetes burnout, complications of diabetes, social support, and self -care behaviors had direct and indirect effect on QOL among patients with T2D. Therefore, this study was aimed to examine the relationship between DHL, distress, burnout, social support, complications of diabetes, self-care behaviors, and QOL among T2D by application Path analysis method.

## Method

### Study design

This Path analysis study was done in 2023 among patients with T2D in Mashhad city, Iran.

### Sample size

The required sample size in this study was calculated using the following formula (0.95% confidence level, the power test of 80%, accuracy/d = 0.06) and according to the previous study in Iran (the standard deviation of QOL = 0.62) [53]. Based considering 10% drop rate, sample size of 929 was calculated.

$$n=\frac{{({z}_{1-\frac{\alpha }{2}} +{z}_{1-\beta })}^{2} {\left(S\right)}^{2} }{{\left(d\right)}^{2}}, n=\frac{\left(7.84\right) {\left(0.62\right)}^{2} }{{\left(0.06\right)}^{2}}=837$$

## Sampling method of participants

Data were gathered from Comprehensive Health Service Centers in Mashhad city by cluster sampling method. Each Comprehensive Health Service Center (*n* = 5) in Mashhad were considered as a cluster and then three clusters were selected by simple random sampling. After that, participants who had inclusion criteria were entered to study. Then, the questionnaires were provided to the participants and questionnaires were answered carefully by self-report. The questionnaires of participants who had not enough literacy was also interviewed and filled out by the researcher. The criteria for the entry of participants in this study were informed consent, having T2D for more than one year, and having an active health file in Mashhad Comprehensive Health Centers. The incomplete questionnaire was also considered as the exclusion criteria of this study.

### Instruments

Data were gathered with seven tools of demographic section, diabetes health literacy scale (DHLS), diabetes distress scale (DDS), diabetes burnout scale, diabetes self-management questionnaire (DSMQ), perceived social support, and diabetes quality of life (DQOL).

#### Demographic section

The questionnaire included questions about age, place of residence, sex, marital status, age of diabetes onset, education level, duration of disease, income status, occupation, and complications of diabetes.

#### DHLS

DHLS was designed and assessed by Lee et al. [54]. The DHLS have 14 items and three subcategories of communication HL (with 3 items), numerate HL (with 4 items) and informational HL (with 7 items). Each item was measure with five-option Likert scale (“Not really” to “Very much”). Psychometric of the Persian version of this tool evaluated in 2022 by Moshki et al., and Cronbach’s alpha coefficient was 0.919 for DHLS [55]. Also, Cronbach’s alpha coefficient for subscales of numerate, communicative, and informational was 0.879, 0.784, and 0.865, respectively [55].

#### DDS

This scale has two parts of Sources of Distress (with 21 items) and Core Level of Distress (with 8 items) and designed by Polonsky et al. [56]. Sources of Distress consists of 7 subscales of, Healthcare Provider, Hypoglycemia, Shame/Stigma, Long-term Health, Interpersonal Issues, Management Demands, and Healthcare Access [56]. Each subscale of Sources of Distress was measured with 3 items. All items were measured with five choice Likert scale (“Not a Problem = 1” to “A Very Serious Problem = 5”) [56]. The validity and reliability of DDS was evaluated by Jafari et al., in Iranian T2D and Cronbach’s alpha coefficients was 0.950 for diabetes distress scale, 0.914 for Core Level of Distress, and 0.920 for Sources of Distress [57].

#### Diabetes burnout scale

This scale has 12 items and three subcategories of Detachment (5 items), Loss of control (3 items), and Exhaustion (4 items) and assessed by Abdoli et al., in 2021. This questionnaire measures diabetes burnout with a 5 -option Likert scale (“Completely agree” to “Completely disagree”). Cronbach’s alpha coefficient of this scale was reported 0.8 [41]. Psychometric of Persian version of this questionnaire was checked by Aslani et al., and Cronbach’s alpha coefficient was reported 0.813 [58].

#### DSMQ

This tool with 16 items examines the self -care behaviors of diabetes in 4 subscales of physical activity, diet control, glucose management and health care. This scale was presented in 2013 by Schmitt et al., and each item is measured with 4 -choice Likert scale (“Applies to me very much” to “Does not apply to me”). In Schmitt study the Cronbach’s alpha coefficient was 0.84 [59]. Psychometric characteristics of this questionnaire was reviewed by Nakhaeizadeh et al., in Iranian people and Cronbach’s alpha coefficient was 0.82 [60].

#### Perceived social support

This tool consists of 6 questions designed for the status of perceived social support of patients with diabetes by Hsiao [61]. The questions were evaluated using a 5 -option Likert scale (Completely agree to completely disagree). The scoring range is between 6 and 30 and the higher score indicates a higher perceived social support [61]. The validity and reliability of perceived social support were investigated in this study and the amount of Cronbach’s elephant was 0.819.

#### DQOL

The design and psychometrics of the DQOL were carried out in 2004 by Burroughs et al. [62]. This questionnaire consists of 15 items and the first 8 questions were measured with 5 choice Likert scale (“Completely not satisfied” to “Completely satisfied”) and the second 7 items were measured with 5 choice Likert scale (Never to Always). The validity and reliability of DQOL were also evaluated among Iranian population and Cronbach’s alpha coefficient was 0.75 [63].

### Statistical analysis

The software of SPSS version 24 was used to analysis data at a significant level less than 0.05. The comparison between variables was done with the tests of Independent-samples t-tests, Chi-square, one way ANOVA, and Pearson correlation. The direct paths and indirect paths between variables were evaluated with AMOS software version 24. In Path analysis, relationship between variables of DHL, social support, diabetes distress, diabetes burnout, complications of diabetes, and self- care behaviors of DQOL was assessed. To assessed the final Path model, the goodness of fit indices of CFI (more than 0.9), GFI (more than 0.9), RMSEA (less than 0.08), RFI (more than 0.9), NFI (more than 0.9), IFI (more than 0.9), AGFI (more than 0.9), TLI (more than 0.9) were used [64–67].

## Results

The response rate of participants was 88.26% and data were analyzed among 820 participants. Most patients were female (*n* = 483, 58.9%), married (*n* = 682, 83.2%), housewife (*n* = 416, 50.7%), had elementary education level (*n* = 238, 29%), and 57.9% (*n* = 475) of participants reported that obtained information related to mental illness. More demographic information and diabetes status information were mentioned in Table 1. In this study, 38% had no complications (*n* = 312), 34.5% only had one complication (*n* = 283), 21% had two complications (*n* = 172), 5.5% had three complications (*n* = 45), 0.7% had four complications (*n* = 6), and only 0.2% had five complications (*n* = 2).

**Table 1:** Frequency the characteristics of demographic variables

Variables				*n* = 820	
				*n*	*%*
Sex	Male			321	39.1
	Female			483	58.9
	Missing information			16	2
Age group	18–27			14	1.7
	28–37			32	3.9
	38–47			98	12
	48–57			206	25.1
	58–67			278	33.9
	68 and more than			173	21.1
	Missing information			19	2.3
Marital status	Married			682	83.2
	Single			75	9.1
	Divorced			35	4.3
	Missing information			28	3.4
Occupation	Housewife			416	50.7
	Employed			47	5.7
	Retired			155	18.9
	Self-employed			103	12.6
	labor			44	5.4
	Unemployed			20	2.4
	Missing information			35	4.3
Education level	Illiterate			132	16.1
	Elementary			238	29
	Secondary			107	13
	High school			67	8.2
	Diploma			135	16.5
	Associate Degree			35	4.3
	Bachelor’s degree			62	7.6
	Master’s degree and more			21	2.6
	Missing information			23	2.8
Economic status	Good			91	11.1
	Medium			486	59.3
	Weak			191	23.3
	Missing information			52	6.3
Get information related to mental illness	Yes			475	57.9
	No			297	36.2
	Missing information			48	5.9
Sources of obtaining health information	Physician/ Health care providers	Yes		419	51.1
		No		401	48.9
	Internet	Yes		136	16.6
		No		684	83.4
	Newspapers/ magazines	Yes		15	1.8
		No		805	98.2
	Friends and acquaintances	Yes		143	17.4
		No		677	82.6
	Book	Yes		35	4.3
		No		785	95.7
	Radio, television and satellite	Yes		191	23.3
		No		629	76.7
	I do not know	Yes		29	3.5
		No		791	96.5
Sources of obtaining information related to mental illness	Physician/ Health care providers	Yes		254	31
		No		566	69
	Psychologist/Psychiatrist	Yes		88	10.7
		No		732	89.3
	Friends and acquaintances	Yes		143	17.4
		No		677	82.6
	Book	Yes		74	9
		No		746	91
	Internet	Yes		132	16.1
		No		688	83.9
	Radio, television and satellite	Yes		185	22.6
		No		635	77.4
	All items above	Yes		63	7.6
		No		757	92.4
Which method do you use to treat your diabetes	Insulin	Yes		148	18
		No		672	82
	Pills and drugs	Yes		661	80.6
		No		159	19.4
	Exercise and physical activity	Yes		166	20.2
		No		654	79.8
	Diet	Yes		235	28.7
		No		585	71.3
	Herbal Medicines	Yes		105	12.8
		No		715	87.2
	All cases	Yes		16	2
		No		804	98
	None	Yes		4	0.4
		No		816	98.6
Refer to a health professional for mental-psychological	Yes			163	19.9
	No			631	77
	Missing information			26	3.2
To which specialist you have been referred for psychiatric problems	Psychologist/ Psychiatrist	Yes		103	12.6
		No		717	87.4
	Physician	Yes		42	5.1
		No		778	94.9
	Nurse	Yes		4	0.5
		No		816	99.5
	Counselor	Yes		14	1.7
		No		806	98.3
	Health care providers	Yes		46	5.6
		No		775	94.4
How helpful to visit the health professional for mental-psychological?	Very useful			31	3.8
	Useful			89	10.9
	Low effect			30	3.7
	Very low effect			10	1.2
	Effectless			4	0.5
	I have no idea			9	1.1
	Missing information			647	78.9
Complications of diabetes	Eye complications		Yes	137	16.7
			No	683	83.3
	Heart complications such as hypertension		Yes	292	35.6
			No	528	64.4
	Kidney complications		Yes	32	3.9
			No	788	96.1
	Wound in one leg		Yes	11	1.3
			No	809	98.7
	Wound in two legs		Yes	4	0.5
			No	816	99.5
	Amputations		Yes	3	0.4
			No	817	99.6
	Hyperlipidemia		Yes	317	38.7
			No	503	61.3

Results of relationship between demographic variables with DHL, diabetes distress, diabetes burnout, social support, and self-care behaviors were mentioned in Table 2. Based on the results, there was a significant relationship between variables of sex, education level, economic status, occupation status, and get information related to mental illness with DHL. There was a significant relationship between variables of education level, economic status, and occupation status with self-care behaviors. Also, there was a significant relationship between education level, occupation status, economic status with DQOL (Table 2).

**Table 2:** Relationship between demographic variables with DHL, diabetes distress, diabetes burnout, social support, self-care behaviors, and DQOL

Variables		*Mean (SD)*											
		**DHL**	P-value	**Diabetes distress**	P-value	**Diabetes burnout**	P-value	**Social support**	P-value	**Self-care**	P-value	**DQOL**	P-value
Sex*	Men	44.07(12.37)	0.001	68.47(25.27)	0.418	31.27(7.52)	0.549	21.97(4.90)	0.026	43.42(5.51)	0.415	53.98(8.60)	0.984
	Women	41.12(11.74)		69.99(26.52)		31.60(7.73)		21.19(4.85)		43.10(5.42)		54.00(8.44)	
Age group**	18–27	51.34 (11.31)	< 0.001	79.06(36.06)	0.371	34.39(9.43)	0.350	20.42(4.20)	0.571	43.17(5.47)	0.913	56.04(11.37)	0.861
	28–37	48.23(13.52)		73.73(26.07)		31.62(6.98)		20.65(6.29)		43.17(5.68)		54.41(8.11)	
	38–47	47.91(10.23)		66.28(27.55)		30.29(7.03)		21.70(5.04)		43.33(5.58)		54(9.01)	
	48–57	42.82(12.51)		68.81(26.46)		31.36(7.53)		21.65(4.84)		42.99(5.69)		53.87(8.53)	
	58–67	40.45(11.16)		68.54(24.91)		31.42(7.80)		21.26(4.85)		43.09(5.33)		53.87(8.27)	
	68 and more than	39.19(11.92)		71.15(25.53)		32.09(7.73)		21.90(4.61)		43.62(5.12)		53.49(8.45)	
Marital status**	Married	42.43(11.85)	0.507	68.28(25.76)	0.007	31.22(7.63)	0.019	21.64(4.98)	0.075	43.25(5.52)	0.809	54.32(8.53)	0.061
	Single	41.61(13.26)		74.35(27.49)		33.17(7.44)		20.45(4.29)		43.26(4.40)		52.85(8.17)	
	Divorced	40.24 (10.74)		79.96(20.80)		33.87(6.86)		20.59(3.80)		42.65(4.84)		51.44(5.46)	
Education level**	Illiterate	32.09(9.40)	0.000	71.66(25.83)	0.003	32.87(7.44)	0.000	21.31(4.25)	0.925	41.37(4.87)	0.000	57.74(8.78)	0.011
	Elementary	37.98(9.56)		68.05(25.24)		31.94(7.47)		21.79(4.65)		42.99(5.10)		53.25(8.02)	
	Secondary	43.62(9.89)		70.84(26.21)		32.26(8.11)		21.33(4.82)		42.93(4.82)		53.89(8.17)	
	High school	44.15(10.96)		78.82(24.78)		32.20(7.96)		21.04(5.19)		43.51(4.82)		52.66(7.48)	
	Diploma	47.66(10.28)		69.26(25.14)		31.34(7.05)		21.43(5.43)		44.43(5.38)		55.16(8.48)	
	Associate Degree	51.85(11.50)		61.76(24.94)		27.83(5.14)		21.89(5.16)		45.46(5.77)		57.52(7.38)	
	Bachelor’s degree	54.04(8.40)		61.31(27.47)		27.30(7.70)		21.61(5.06)		44.18(6.92)		55.48(10.37)	
	Master’s degree and more	56.82(9.98)		62.65(23.68)		28.57(5.17)		22.21(4.14)		46.24(6.88)		55.98(9.01)	
Occupation**	Housewife	39.83(11.36)	0.000	70.13(26.09)	0.006	31.60(7.78)	0.096	21.33(4.62)	0.022	43.07(5.36)	0.001	53.97(8.34)	0.004
	Employed	51.85(11.14)		70.88(26.71)		30.02(7.33)		20.84(4.95)		43.47(4.53)		53.01(7.87)	
	Retired	44.80(11.14)		65.32(25.11)		30.54(7.74)		22.68(5.25)		44.54(5.97)		55.73(8.50)	
	Self-employed	45.04(12.88)		68.69(25.50)		31.61(7.48)		21.65(4.93)		43.26(5.78)		53.41(8.86)	
	labor	37.29(11.79)		72.54(23.75)		33.78(5.96)		20.48(4.73)		40.47(3.82)		51.28(7.72)	
	Unemployed	38.87(14.17)		88.89(28.22)		33.31(6.92)		20.82(4.07)		42.23(4.80)		49.68(9.30)	
Economic status**	Good	46.47(12.15)	0.000	57.98(22.21)	0.000	27.58(7.77)	0.000	21.46(5.32)	0.026	45.76(5.85)	0.000	57.47(7.69)	0.000
	Medium	44.21(11.50)		66.91(24.43)		30.90(7.24)		21.75(4.85)		43.50(5.39)		54.94(8.09)	
	Weak	35.98(11.03)		81.29(28.80)		35.12(7.24)		20.62(4.74)		41.57(5.07)		49.73(8.36)	
Get information related to mental illness*	Yes	44.81(11.71)	0.000	70.41(26.09)	0.284	31.51(7.51)	0.878	22.20(4.41)	0.000	43.51(5.75)	0. 093	53.85(8.49)	0.768
	No	38.15(11.37)		68.35(25.71)		31.42(7.77)		20.47(5.42)		42.85(5.00)		54.04(8.42)	

* Independents sample T-test, ** One-way ANOVA

Results of correlation between variables of DHL, social support, diabetes distress, diabetes burnout, self- care behaviors, and DQOL were mentioned in Table 3. In this study, a negative and significant correlation was found between diabetes distress (*p* < 0.001, *r* = − 0.653) and diabetes burnout (*p* < 0.001, *r* = − 0.535) with DQOL. Also, a positive and significant correlation was found between DHL (*p* < 0.001, *r* = 233), social support (*p* < 0.001, *r* = 0.220), and self- care behaviors (*p* < 0.001, *r* = 0.369) with DQOL (Table 3).

**Table 3:** Pearson correlation between variables

Variables	DHL	Diabetes distress	Diabetes burnout	Social support	Self-care behaviors
DHL**	1				
Diabetes distress	− 0.137^*^	1			
Diabetes burnout	− 0.232^*^	0.584^*^	1		
Social support	0.293^*^	− 0.184^*^	− 0.188^*^	1	
Self-care behaviors	0.356^*^	− 0.266^*^	− 0.360^*^	0.304^*^	1
DQOL***	0.233^*^	− 0.653^*^	− 0.535^*^	0.220^*^	0.369^*^

* Correlation is significant at the <0.001 level (2-tailed)

** Diabetes health literacy

*** Diabetes quality of life

**Fig. 1:**
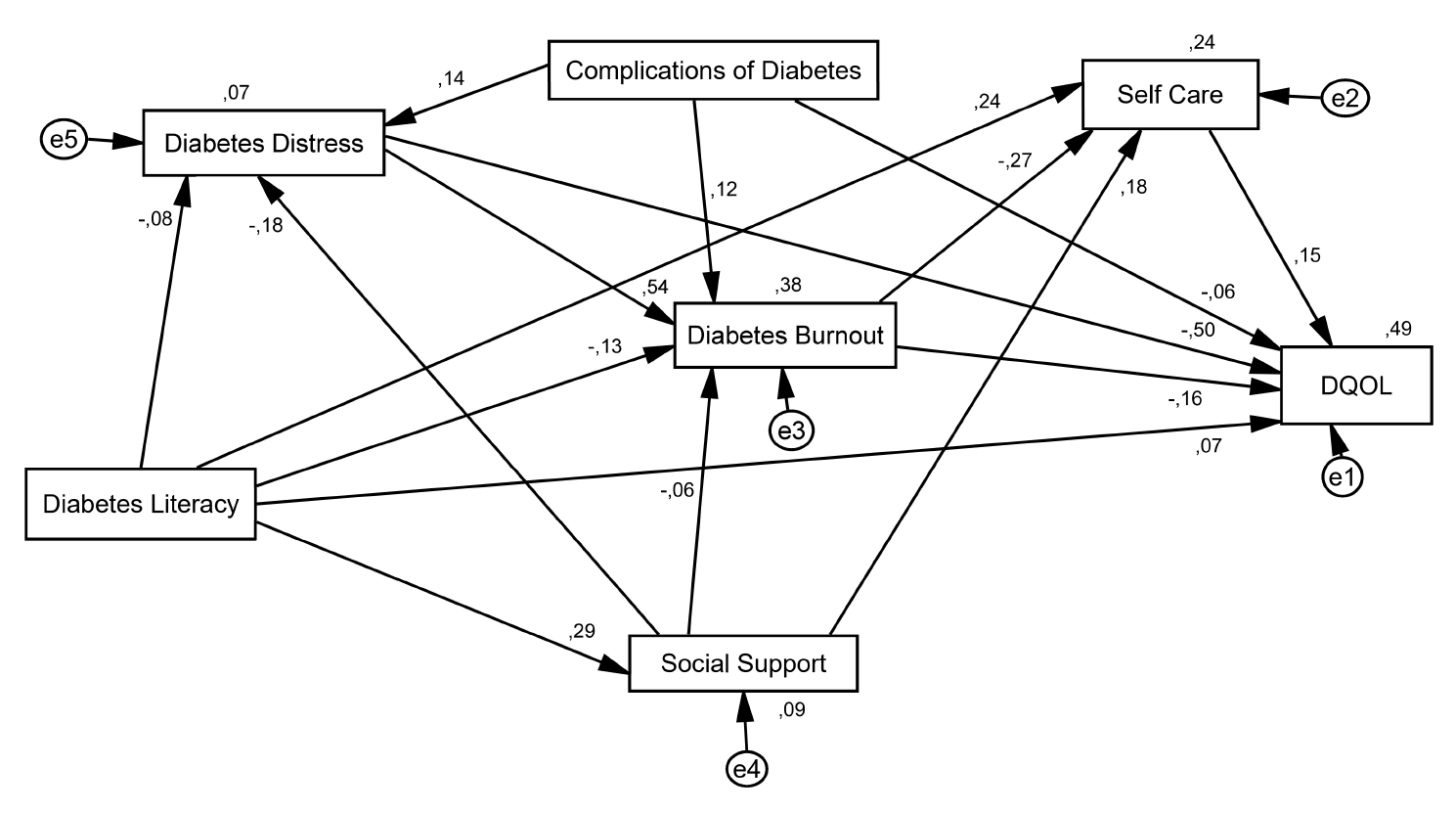
Direct and indirect paths between variables in prediction of diabetes burnout (R^2^ = 38%), self-care behaviors (R^2^ = 24%), and DQOL (R^2^ = 49%)

According the results of confirmatory factor analysis, goodness of fit indices (for example: RMSEA = 0.070, CFI = 0.984, AGFI = 0.952) confirmed the paths between variables (Table 4). Standardized total effects, standardized indirect effects, and standardized direct effects between variables mentioned in Table 5. The variables of DHL, social support, diabetes distress, and complications of diabetes predicted 38% variance in diabetes burnout (R^2^ = 0.38) (Fig. 1). Greatest impact on diabetes burnout was related to diabetes distress (estimate total effect = 0.539) (Table 5). The variables of DHL, social support, diabetes distress, complications of diabetes, and diabetes burnout predicted 24% variance in self- care behaviors (R^2^ = 0.24) (Fig. 1). Greatest impact on self- care behaviors was related to DHL (estimate total effect = 0.354) (Table 5). The variables of DHL, social support, diabetes distress, diabetes burnout, complications of diabetes, and self- care behaviors predicted 49% variance in DQOL (R^2^ = 0.49) (Fig. 1). In this study, greatest impact on DQOL was related to variables of diabetes distress (estimate total effect = -0.613), DHL (estimate total effect = 0.225), diabetes burnout (estimate total effect = -0.202), complications of diabetes (estimate total effect = − 0.173), social support (estimate total effect = 0.149), and self -care (estimate total effect = 0.149), respectively (Table 5).

**Table 4:** The model fit indicators of path model

Goodness of fit indices	Confirmatory factor analysis	Acceptable value
X^2^	29.911	-
df	6	-
X^2^/df	4.985	< 5
P-value	0.000	> 0.05
CFI	0.984	> 0.9
GFI	0.991	> 0.9
RMSEA	0.070	< 0.08
RFI	0.920	> 0.9
NFI	0.981	> 0.9
AGFI	0.952	> 0.9
IFI	0.985	> 0.9
TLI	0.935	> 0.9

**Table 5:** Direct and indirect paths between variables

Determinants or Predictors	Standardized effects		
	Standardized direct effects	Standardized indirect effects	Standardized total effects
DHL → Diabetes distress	-0.078**	-0.052*	-0.130**
DHL → Diabetes burnout	-0.134*	-0.089*	-0.223*
DHL → Social support	0.293*	-	0.293*
DHL → Self-care behaviors	0.240*	0.114*	0.354*
DHL → DQOL	0.071**	0.154*	0.225*
Diabetes distress → Diabetes burnout	0.539*	-	0.539*
Diabetes distress → Self-care behaviors	-	-0.146*	-0.146*
Diabetes distress → DQOL	-0.504*	-0.109*	-0.613*
Diabetes burnout → Self-care behaviors	-0.270*	-	-0.270*
Diabetes burnout → DQOL	-0.162*	-0.040*	-0.202*
Social support → Diabetes distress	-0.178*	-	-0.178*
Social support → Self-care behaviors	0.183*	0.043*	0.226*
Social support → Diabetes burnout	-0.063**	-0.096*	-0.159*
Social support → DQOL	-	0.149*	0.149*
Complications of diabetes → Diabetes distress	0.140*	-	0.140*
Complications of diabetes → Diabetes burnout	0.118*	0.075*	0.193*
Complications of diabetes → Self-care behaviors	-	-0.052*	-0.052*
Complications of diabetes → DQOL	-0.063**	-0.110*	-0.173*
Self-care behaviors → DQOL	0.149*	-	0.149*
Total causal effect	3.185/4.414	1.229/4.414	4.414
Percantage of direct and indirects effects	72%	28%	100

DQOL: Diabetes quality of life, DHL: Diabetes health literacy, *P < 0.001, **P < 0.05

## Discussion

This study aimed to examine the relationship between DHL, distress, burnout, social support, complications of diabetes, self-care behaviors, and QOL among T2D by application Path analysis method. In general, the results showed that variables of DHL, social support, diabetes distress, diabetes burnout, complications of diabetes, and self -care behaviors were able to predict 49% of the variance of DQOL. These results showed that people with higher DHL, low level of burnout and distress, more social support, low complications, and better self -care behaviors had better DQOL. These findings suggest that enhancing DHL, reducing burnout and distress, increasing social support, prevention of complications, and promoting self-care behaviors can contribute to better health-related QOL. Also, ALSharit conducted a study aimed at determining the effect of HL on blood glucose control, self -management and QOL among T2D, and the results showed that self -care was a mediator variable between HL and DQOL [68]. In the present study, the variables of social support, diabetes distress, and diabetes burnout were specifically examined, in addition to DHL and self-care. Unlike in the ALSharit study, the role of these variables in the QOL was not considered [68].

The results of this study showed that DHL and diabetes burnout had the greatest effect on the self -care behaviors. DHL had positive and direct effect on self -care, meaning that patients with higher HL had better self -care. Patients with higher HL levels are more likely to engage in effective self-care practices, such as adhering to treatment plans, better management of condition, and making informed health decisions [69]. Previous studies have also shown that low HL is associated with a decrease in self -care behaviors [49, 70]. Burnout also had negative and direct effect on self -care behaviors, meaning that people with less burnout had better self -care behaviors. Burnout in diabetes is caused by fatigue from performing continuously self -care behaviors. This can lead to not performing self -care behaviors [71]. In a study, the lack of tendency to perform self -care behaviors were repeated symptoms of diabetes burnout and most patients lost their diabetes control [72].

The results of path analysis on DQOL have shown that diabetes distress, DHL, diabetes burnout, complications of diabetes, social support, and self -care had the greatest impact on DQOL, respectively. Accordingly, diabetes distress and diabetes burnout had negative and direct effect on the DQOL, meaning that patients with distress and burnout had less DQOL. Diabetes burnout and diabetes distress is a combination of emotions and acts that are related to fatigue to incuriosity and are associated with the feeling of despair [44]. Krstović-Spremo showed a relationship between diabetes burnout and diabetes distress with QOL in patients with type 1 diabetes and hypertension [73]. Also, distress and burnout were higher in people with type 1 diabetes than people with hypertension [73]. While Krstović-Spremo et al.‘s study focused on type 1 diabetes patients, this study explored the role of additional variables on the QOL of patients with T2D [73].

Regarding DHL, the results also showed that DHL has a positive and direct effect on DQOL. This result showed that patients with higher DHL had better DQOL. Patients with adequate HL are more empowered to navigate the complexities of their conditions, leading to better health outcomes and a higher QOL. HL increases health promotion behaviors, reduces disease complications and improves DQOL [74]. Patients with low HL may pay little attention to their health and therefore choose unhealthy behaviors that reduce their DQOL [74]. Results an study has also shown that inadequate HL is associated with less use of preventive health services, which also reduces the DQOL in these patients [75]. In Esen study, results showed that patients with high HL are more compatible with physician recommendations and less complications occur in these patients and QOL of patients would be better if there are no complications [76]. Also, the results of some studies showed that HL predicts DQOL and that adequate HL has an important factor in improving the DQOL [77, 78]. In contrast to all the above studies that have exclusively focused on the role of HL on QOL, this study investigates the role of HL on the QOL of patients with T2D, considering additional relevant variables such as social support, diabetes distress, and job burnout. The study also examines the relationship between diabetes and self-care.

Social support also had positive and indirect effect on DQOL, so that social support reduced burnout and improved self -care behaviors in patients with diabetes. This result showed that patients who received more social support felt less burnout and had better DQOL. In a study, results showed that social support adjusts the effects of stress related to diabetes management, facilitates effective coping, reduces burnout, and ultimately improves DQOL in patients with diabetes. In addition, social support has improved the DQOL in patients by influencing self -care behaviors [23]. Also results a systematic review showed that patients with diabetes with more perceived social support were more likely to follow self-care behaviors and had better DQOL [79].

In this study, according to the results of path analysis, self -care behaviors had positive and direct effect on the DQOL. This result showed that patients with better self -care management had better DQOL. Proper self -care behaviors were associated with good control of blood sugar, reduced complications and improved DQOL. Findings confirmed that self-care behaviors were also identified as the most important predictor of QOL in diabetes [48]. Also, Lee study showed that self -care behaviors were very important for the relationship between HL and QOL in patients with diabetes [80]. This study was conducted with high sample size among T2D and used valid and reliable tools. One of the weaknesses was that only the relationships between variables can be measured.

### Strengths and limitation

One strength of this research was the use of a large sample size, which helps to minimize measurement biases. In this study, we faced some limitations, such as, data collection was conducted using a self-reporting method, which could potentially impact the individual reporting of data. Additionally, the use of questionnaires and the challenge of fully generalizing the results to other societies and cultures are additional limitations of this research.

## Conclusion

The results of this study helped to understand more effective variables in predicting DQOL. Diabetes burnout, distress and complications as three potential factors had direct and indirect negative effects on DQOL. However, high DHL and strong social support can help modify and neutralize the negative effects of distress and burnout, as well as promote diabetes self -care behaviors, and ultimately promote the DQOL.

Increasing DHL level of people makes the patients aware of their disease, act more committed to medical orders, and have more self-care, and finally, they will have a better QOL. Therefore, to improve QOL in patients with diabetes, health care providers must develop interventions that increase the HL of patients with diabetes. Because DHL can help enhance self -care skills and create supportive networks, and ultimately improve QOL in diabetes by reducing distress and burnout.

## Data Availability

No datasets were generated or analysed during the current study.

## Abbreviations

DQOL: Diabetes quality of life
QOL: Quality of life
DHL: Diabetes health literacy
HL: Health literacy
DDS: Diabetes distress scale
DSMQ: Diabetes Self-Management Questionnaire
AGFI: Adjusted goodness of fit index
X^2^: Chi-square
Df: Degree of freedom
IFI: Incremental fit index
NFI: Normed fit index
RMSEA: Root mean square error of approximation
CFI: Comparative fit index
TLI: Tucker lewis index
GFI: Goodness of fit index
RFI: Relative fit index
